# Patients with periodontitis exhibit persistent dysbiosis of the gut microbiota and distinct serum metabolome

**DOI:** 10.1080/20002297.2025.2499284

**Published:** 2025-05-08

**Authors:** Eiji Miyauchi, Kyoko Yamazaki, Yuuri Tsuboi, Takako Nakajima, Shigeru Ono, Kentaro Mizuno, Naoki Takahashi, Kentaro Imamura, Hidetoshi Morita, Nobuaki Miura, Shujiro Okuda, Jun Kikuchi, Nobuo Sasaki, Hiroshi Ohno, Kazuhisa Yamazaki

**Affiliations:** a Laboratory for Intestinal Ecosystem, RIKEN Center for Integrative Medical Sciences (IMS), Yokohama, Japan; b Laboratory of Mucosal Ecosystem Design, Institute for Molecular and Cellular Regulation, Gunma University, Maebashi, Japan; c Division of Gastroenterology and Hepatology, Department of Internal Medicine, University of Michigan, Ann Arbor, MI, USA; d RIKEN Center for Sustainable Resource Science, Yokohama, Japan; e Nakajima Dental Clinic, Niigata, Japan; f Ono Dental Clinic, Niigata, Japan; g Mizuno Dental Clinic, Niigata, Japan; h Division of Periodontology, Department of Oral Health Science, Faculty of Dental Medicine, Hokkaido University, Sapporo, Japan; i Department of Periodontology, Tokyo Dental College, Chiyoda-Ku, Japan; j Laboratory of Animal Applied Microbiology, Graduate School of Environmental and Life Science, Okayama University, Okayama, Japan; k Division of Bioinformatics, Niigata University Graduate School of Medical and Dental Sciences, Niigata, Japan; l Medical AI Center, School of Medicine, Niigata University, Niigata, Japan

**Keywords:** Periodontitis, saliva, gut, microbiome, metabolome

## Abstract

**Objectives:**

Animal studies suggest that periodontopathic bacteria induce gut dysbiosis and related pathology, possibly connecting periodontitis to non-oral diseases. However, the effects on the gut ecosystem in periodontitis patients are not fully understood.

**Methods:**

We conducted a comprehensive analysis of the salivary and gut microbiota using 16S rRNA sequencing in periodontitis patients before and after treatment, comparing them to healthy participants. Serum metabolites were also analyzed.

**Results:**

Periodontitis patients showed high alpha diversity in both salivary and gut microbiota with a strong correlation. Significant differences were also observed in the gut microbiota composition between patients before treatment and healthy participants, irrespective of the ectopic colonization of periodontitis-associated bacteria in the gut. Co-abundance group analysis demonstrated that the gut microbiota of healthy participants was enriched with short-chain fatty acid producers. Changes in the gut microbiota coincided with alterations in the serum metabolite profile. While periodontal therapy improved salivary microbiota, it did not significantly affect gut microbiota.

**Conclusions:**

Gut dysbiosis of periodontitis patients may impact systemic metabolite profiles. Given that periodontal therapy alone did not substantially improve the gut microbiota, adjunctive strategies targeting the gut microbiome may be effective in reducing the risk of periodontitis-associated diseases.

## KEY MESSAGES

- Patients with periodontitis demonstrated gut microbiota dysbiosis, characterized by a decreased proportion of short-chain fatty acid-producing bacteria.
- Changes in the microbiota were accompanied by alterations in the serum metabolome.
- Although periodontal therapy improved the salivary microbiota, it had little effect on gut microbiota composition, highlighting the need for adjunctive interventions targeting the gut microbiota to mitigate systemic risks associated with periodontitis.

## Introduction

There is an increasing amount of evidence suggesting that periodontal disease is associated with an increased risk for various metabolic, inflammatory, and autoimmune diseases, such as type-2 diabetes, atherosclerotic vascular diseases, and rheumatoid arthritis [1].

Although the pathogenesis of each disease is different, a common pathological feature of these diseases is inflammation. Thus, bacteremia, endotoxemia, and the invasion of locally produced proinflammatory cytokines into the systemic circulation are considered the causal mechanisms by which periodontal disease increases the risk for systemic diseases [1]. Consistent with this hypothesis, numerous studies have demonstrated the presence of periodontopathic bacterial DNA in various tissues and organs [2–4] and an elevated level of proinflammatory cytokines, particularly interleukin-6, in the blood [5–7]. Dental procedures such as scaling and root planing (SRP) and even tooth brushing are reported to induce transient bacteremia [8]. However, there are no reports on bacteremia in patients with periodontitis in the absence of any procedure. In addition, no bacterial DNA was detected in the blood of patients with periodontitis one day after undergoing one-stage full-mouth SRP [9]. There is no direct evidence that the bacterial DNA detected in the tissues such as blood vessels and the liver is derived from bacteria that migrate from periodontal pockets into the body or that the cytokines detected in the blood are produced in the periodontal disease lesions. These data suggest that there may be other pathological mechanisms in addition to bacteremia/endotoxemia and the overflow of inflammatory mediators from the lesion for the spread to other parts of the body.

Recently, we proposed a novel hypothesis regarding the link between periodontal disease and systemic diseases based on the mouth – gut connection. We demonstrated that oral administration of *Porphyromonas gingivalis*, a representative periodontopathic bacterium, can change the gut microbiota composition (dysbiosis), which is associated with impaired gut barrier function, resulting in endotoxemia and subsequent inflammation of the liver and adipose tissue [10,11]. We and others confirmed that *P. gingivalis*-associated intestinal pathogenic mechanisms are involved in the worsening of the disease [12–15]. Furthermore, oral dysbiosis induced by ligature-induced periodontitis also promotes colitis, cognitive decline, and multiple sclerosis-like symptoms via gut dysbiosis [16–18]. Thus, gut dysbiosis likely plays a role in the pathological interaction between periodontitis and systemic diseases. Besides gut dysbiosis itself, a large part of the effect of gut dysbiosis on systemic health is attributable to microbial metabolites. In particular, amino acid and lipid metabolites can influence the progression of chronic inflammation, autoimmune diseases, cardiovascular disease, and metabolic syndrome [19].

However, human studies have recently been initiated, and research to extend the application of the results of animal studies to humans is lacking. Early studies demonstrated lower alpha diversity and changes in several bacterial phyla in the gut microbiome of patients with chronic periodontitis than in that of healthy participants [20]. The same group reported that the profiles of oral (salivary) and gut microbiota are different between patients with periodontitis and healthy participants [21]. Although there are several related studies, all of them, including those described above, are exploratory and preliminary [22,23]. Therefore, this study investigated the profile of the salivary and gut microbiota in patients with periodontitis before and after treatment and compared them with those of otherwise healthy participants. We applied a bioinformatics approach to the metabolomics of serum samples to gain insights into the unknown mechanism connecting periodontitis and extraoral diseases.

## Materials and methods

### Clinical study design

Patients with Stage III, Grade B periodontitis were recruited from outpatients at Niigata University Medical and Dental Hospital, Tokyo Dental College Suidobashi Hospital, and the cooperating private dental clinics from April 2014 to December 2019. A periodontitis case is defined if 1) Interdental clinical attachment level (CAL) is detectable at ≥2 non-adjacent teeth, or 2) Buccal or oral CAL ≥3 mm with pocketing >3 mm is detectable at ≥2 teeth [24]. The inclusion criteria included 1) having treatment-naïve or extensive recurrent periodontitis; 2) good general health without any remarkable history of disease except for well-controlled hypertension, dyslipidemia, or diabetes; and 3) the presence of at least 20 teeth. The exclusion criteria were 1) use of antibiotics in the past 3 months, 2) pregnancy or possibly pregnancy and 3) other complications affecting the study outcome such as obesity and receiving probiotics, which are reported to be associated with gut dysbiosis.

Periodontally healthy control participants were recruited from the staff members of Niigata University Medical and Dental Hospital, and patients who presented for reasons other than periodontal treatment met the above criteria, except for the first inclusion criterion.

Of the 33 patients recruited, three were excluded from the analysis as all the necessary data could not be obtained. A further 7 patients dropped out during the follow-up period, thus 23 patients were analyzed after periodontal treatment. For the control subjects, the necessary analyses were carried out for all 23 recruited individuals.

After initial screening and diagnosis, periodontal health-related metrics (plaque control record [PCR], probe depth [PD], clinical attachment loss [CAL], bleeding on probing [BOP], periodontal epithelial surface area [PESA], and periodontal inflammatory surface area [PISA]) were recorded by trained dentists. PCR represents the extent of dental plaque deposition. PD, CAL, and PESA represent the degree of periodontal tissue destruction. BOP and PISA represent the degree of gingival inflammation. Initial periodontal therapy consisted of mechanical plaque control, together with SRP under local anesthesia. The effect of the initial periodontal therapy was evaluated, and periodontal surgery was implemented in the case of residual periodontal pockets. The number of sites at which surgical procedures were performed varied among patient. After completion of the procedures, the patients were re-evaluated and followed up monthly or every 3 months depending upon individual requirements. Samples were collected at least 1 month after the completion of active therapy (SRP or periodontal surgery), and healing was confirmed.

### Collection of biospecimens

Unstimulated saliva and fecal samples were collected immediately after the initial examination from all participants and re-evaluation examination following the completion of periodontal therapy from patients with periodontitis.

Fresh saliva and feces were collected and stored under anaerobic conditions in an AnaeroPack™ (Mitsubishi Gas Chemical Co. Inc., Tokyo, Japan) at 4 °C and transported to the laboratory within 24 h of collection. At the laboratory, samples were immediately frozen using liquid nitrogen and stored at  − 80°C until further analyses.

The isolation of microbial DNA and 16S rRNA sequencing were performed at Okayama University and MyMetagenome Co., Ltd. (now Metagenome Co., Ltd., Tokyo, Japan), and data analysis from human saliva samples was performed as described in a previous study [25]. Briefly, the samples were sequentially treated with 100 μg/mL RNase A (Invitrogen, USA), 3 mg/mL of lysozyme (Sigma-Aldrich, USA), 2,000 U/mL of purified achromopeptidase (Wako, Japan), and 1 mg/mL of proteinase K (Nacalai, Japan), and the DNA was subsequently purified with phenol:chloroform:isoamyl alcohol (25:24:1). The V1–V2 region of the 16S rRNA gene was amplified using barcoded primers (27Fmod/338 R), and the pooled PCR products were sequenced on a MiSeq platform using a MiSeq Reagent Kit v3 (600 cycles, Illumina, USA).

### 16S rRNA gene analysis

The filtered and denoised reads were processed with DADA2 (v1.22.0) [26] in R (v4.1.2) to generate the ASVs according to the DADA2 pipeline v1.8 (https://benjjneb.github.io/dada2/tutorial_1_8.html). The taxonomy of ASVs was assigned against the SILVA database v138.1 [27], followed by the removal of ASVs corresponding to mitochondria and chloroplasts. The Phyloseq package (v1.38.0) [28] was used for downstream analyses, including alpha and beta diversities. For alpha diversity indices and the Venn diagram of ASVs, count data were rarefied to the minimum read count (4,734 reads/sample). Differentially abundant genera between groups were detected using the ANCOMBC package (v1.4.0) [29] with default parameters. The correlation analysis between salivary and fecal bacteria was performed using relative abundance values with the microbiome package (v1.16.0). The relative abundance table of fecal samples at the genus level (>0.005% across all samples) was used for the clustering of the participants and co-abundance group (CAG) analysis, according to a previous report [30]. Hierarchical clustering of the participants was performed with distance 1-Pearson correlation coefficients and the Ward.D2 method [31]. The CAG was determined by the Ward.D2 method based on Kendall’s tau coefficients. Positive correlations between the genera (Kendall’s tau > 0.3, *P*-value < 0.05) and the relative abundance or overabundance of each genus were depicted as network plots using Cytoscape (v3.9.1). The abundance of KEGG orthologs (KOs) and pathways was predicted with 16S rRNA gene data using PICRUSt2 (v 2.5.0) [32]. Differentially abundant KOs and pathways were identified using default parameters with the ALDEx2 package (v1.26.0) [33].

### Metabolite analysis

Serum samples with 100 mmol/L potassium phosphate buffer (in deuterium oxide containing 1 mmol/L sodium 2,2-dimethyl-2-silapentane-5-sulfonate, pH = 7.0) were measured on an NMR spectrometer (Bruker DRU-700, Bruker Biospin, Rheinstetten, Germany) as described previously [34]. All baseline 1D projections were collected, and the peaks were identified by rNMR as region of interest (ROI) peaks [35]. Metabolite annotations were performed with our standard metabolite database [36,37]. Therefore, the annotated metabolites and unannotated ROI peaks were used for the following computations.

PLS-DA of the serum water-soluble metabolites was performed with log-transformed and autoscaled data using the Metaboanalyst (v5.0). Spearman’s correlations between the peak intensities of metabolites (log-transformed and autoscaled values) and the relative abundance of genera were analyzed with the microbiome package.

Data visualization was performed using the following R packages: ggplot2 (v3.4.1) [38], cowplot (v1.1.1), ggalluvial (v0.12.5), ggVennDiagram (v1.2.2) [39], pheatmap (v1.0.12), and made4 (v1.68.0) [40].

### Quantification of serum proteins

Inflammation and aging-related proteins in serum samples were analyzed with Human Magnetic Luminex® (Luminex Corporation, Austin, TX) Assay (R&D Systems, Minneapolis, MN) at Filgen, Inc. (Nagoya, Japan).

### Statistical analysis

All the statistical analyses, except where otherwise noted, were performed using the rstatix (v0.7.2) and vegan (v2.6.4) packages in R. The normality of the data distribution was tested with the Shapiro – Wilk test. Differences between the three groups were tested using the Kruskal – Wallis test followed by Dunn’s multiple comparison test (for non-normally distributed variables). Two-group comparisons were performed with the two-tailed unpaired Student’s *t*-test (for normally distributed variables) or the two-tailed unpaired Mann – Whitney test (for non-normally distributed variables). Two-tailed paired t-tests were applied to compare the data before and after treatment. Fisher’s exact test was used for differences in group distribution.

Associations between microbial alpha diversity indices (Chao1 and Shannon) and clinical parameters were assessed using Spearman’s rank correlation. Beta diversity was evaluated by principal coordinates analysis (PCoA) based on weighted and unweighted UniFrac distances. Clinical variables were fitted as vectors onto the ordination space using environmental fitting (envfit, vegan v2.6–4), and statistical significance was determined by 999 permutations.

## Results

### Demographic profiles of patients and controls

Thirty patients with periodontitis (mean age 55.6 years) [41] and 23 periodontally healthy controls (mean age 52.5 years) were analyzed. Sampling from seven patients was not completed because of systemic antibiotic use or dropout, resulting in 23 patients after periodontal treatment. The demographics of the participants are shown in Table S1. All periodontal parameters significantly improved after treatment (Table S1). There were no significant differences in age or body mass index among the groups. The sex ratios were not significantly different between the control and patient groups at pre- and posttreatment, implying that differences in sex ratios do not substantially affect the overall results.

### Serum inflammatory markers

To further characterize the periodontitis status, we analyzed the serum high-sensitivity C-reactive protein (hs-CRP) and growth differentiation factor-15 (GDF15) levels. However, hs-CRP did not differ among the groups; although it was greater in the patients before treatment, blood GDF15 levels, which were greater in patients with periodontitis, decreased significantly after successful periodontal treatment (Figure S1).

### Characteristics of the oral microbiota in periodontitis and impact of periodontal treatment

**Figure 1.**
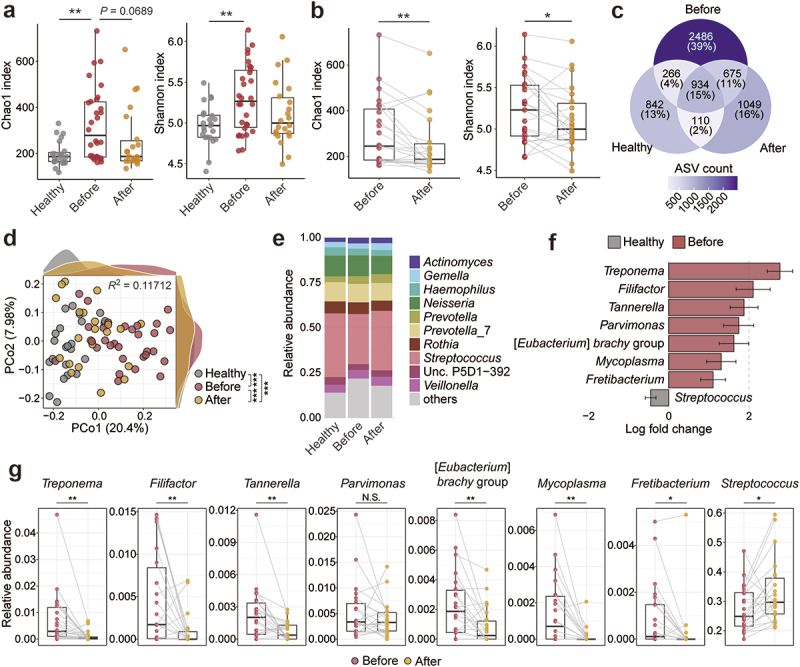
Oral microbiota of healthy participants and patients with periodontal diseases.

To assess the impact of periodontal disease and treatment on the oral microbiota, we performed a 16S rRNA-based microbiota analysis. The Chao1 index and the number of observed ASVs indicated that patients before treatment had increased species richness compared to healthy participants (Figure 1a, Figure S2A). In most patients, the Chao1 index and the number of observed ASVs decreased after periodontal treatment (Figure 1b, Figure S2B). Although the Pielou’s index was comparable, the Shannon index also increased in the patients and decreased after treatment (Figure 1a,b, Figure S2A-B). In addition, a comparison of amplicon sequence variants (ASVs) detected in each group showed that 2,486 ASVs were found only in the patients before treatment (Figure 1c). Principal coordinate analysis (PCoA) based on unweighted UniFrac distances also demonstrated that the oral microbiota of patients before treatment was distinct from that of healthy participants and patients after treatment (Figure 1d). The average relative abundance of each genus indicated that the abundance of *Streptococcus* was low, while that of other minor genera was high in the patients before treatment (Figure 1e). Analysis of Compositions of Microbiomes with Bias Correction (ANCOMBC) also revealed a significant decrease in *Streptococcus* and an increase in periodontitis-associated bacteria in patients before treatment compared to healthy participants (Figure 1f). As expected, periodontal treatment reduced most of these periodontitis-associated bacteria [42] and increased *Streptococcus* (Figure 1e–g). These data clearly showed the altered oral microbiota in patients with periodontal disease and improvement with treatment.

(a) Chao1 and Shannon indices of the salivary microbiota of healthy participants (healthy) and patients before and after treatment. Each point represents an individual. Boxes indicate the median and interquartile range. (b) Effect of periodontal treatment on the Chao1 and Shannon indices. The same participant before and after treatment was connected by a line. (c) Venn diagram showing the shared and unique ASVs (mean abundance > 0.01%) between healthy participants and patients. (d) Principal coordinate analysis of unweighted UniFrac distances. Each point represents a fecal sample. Axes show principal coordinates, with the percentage of variance explained in parentheses. The density plots show the sample distribution along the PCo1 and PCo2 axes. The effect size (*R*^2^) was calculated by Adonis. (e) Bar plot representing the average abundance of the top 10 genera in the salivary samples of healthy participants and patients with periodontal disease before and after treatment. The remainder were labelled ‘others’. (f) Differentially abundant genera (adjusted *P*-value <0.05) between healthy participants and pretreatment patients analyzed by ANCOMBC. (g) Changes in the featured genera in (f) before and after treatment. *p < 0.05, **p < 0.01, ***p < 0.001, with N.S. indicating no statistical significance; Kruskal – Wallis test with Dunn’s test (a), two-tailed paired t-test (b, g), and pairwise Adonis (d).

Interestingly, alpha diversity was significantly and positively correlated with several clinical parameters, including PISA, BOP, and PCR, suggesting a link between the emergence of periodontopathic bacteria and periodontal inflammation (Table 1). Similarly, all clinical parameters demonstrated a significant positive correlation with β-diversity, implicating that the changes in the microbial composition are associated with periodontal pathology (Table 3).

**Table 1.** Correlation between α-diversity in oral microbiota and clinical parameters.

	Chao1 index		Shannon index	
Valuables	*rho*-value	*P*-value	*rho*-value	*P*-value
PPD	0.144	0.217	0.128	0.273
CAL	0.203	0.0813	0.19	0.103
BOP	0.299	0.0091	0.238	0.0393
PISA	0.247	0.0324	0.193	0.0969
PESA	0.087	0.459	0.06	0.607
Number of retained teeth	−0.248	0.0316	−0.242	0.0363
PCR	0.495	0.0000064	0.42	0.000174

Associations between microbial alpha diversity indices and clinical parameters were assessed using Spearman’s rank correlation.

PCR = Plaque control record, PPD = Probing pocket depth, CAL = Clinical attachment level, BOP = Bleeding on probing, PESA = Periodontal epithelial surface area, PISA = Periodontal inflammatory surface area.

### Compositional analysis of gut microbiota

**Figure 2.**
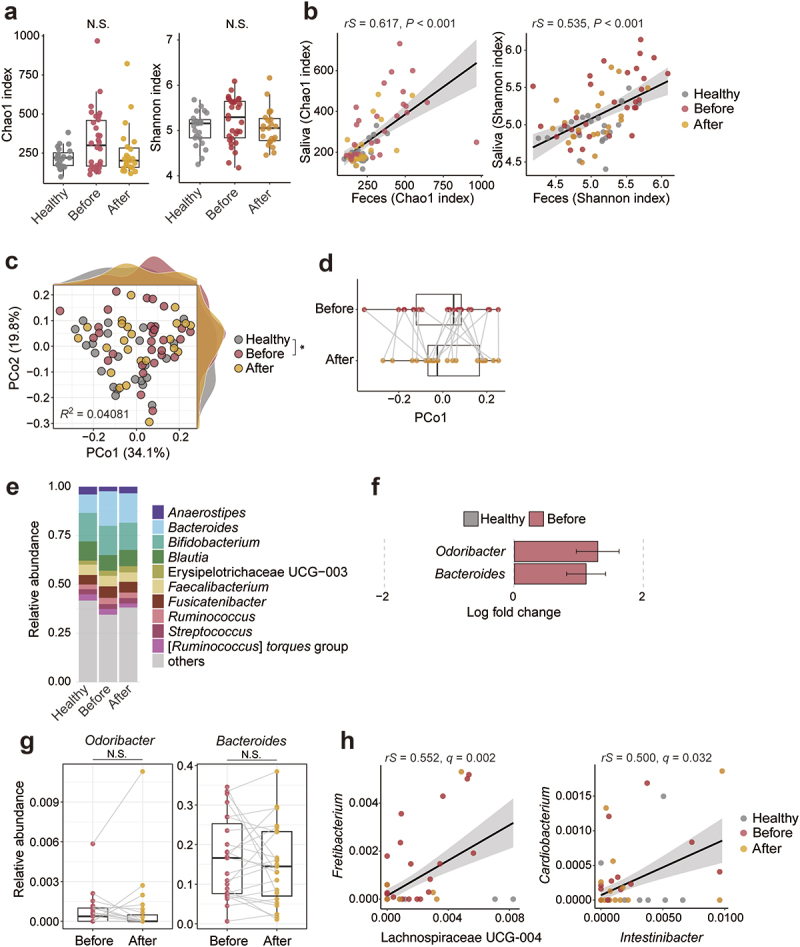
Gut microbiota of healthy participants and patients with periodontal diseases.

Similar to those of the oral microbiota, the gut microbiota of patients before treatment tended to have a greater Chao1 index and number of observed ASVs (Figure 2a, Figure S2C), and positive correlations were observed for these indices of the oral and gut microbiota (Figure 2b, Figure S2D). Similar to the oral microbiota, Pielou’s index of the gut microbiota showed no discernible differences between the groups (Figure S2C-D). The Shannon index of the gut microbiota also exhibited a similar trend and a positive correlation with that of the oral microbiota (Figure 2a,b). Chao1 and Shannon indices were also positively correlated with PCR values (Table 2). PCoA demonstrated significant changes in the microbiota composition of patients before treatment compared to that of healthy participants along the PCo1 axis (Figure 2c). However, no consistent changes in the gut microbiota along the PCo1 axis were observed in the patients after treatment (Figure 2d). The relative abundances of *Odoribacter* and *Bacteroides* were high in the patients before treatment (Figure 2e,f), although the abundances of these genera did not change after treatment (Figure 2g). In addition, the beta diversity of the gut microbiota was correlated with the PPD, CAL, and PESA, clinical parameters showing the severity of periodontitis although the correlation coefficient was low (Table 3). These results indicate that periodontitis induces dysbiosis of the gut microbiota but that periodontal treatment does not reverse it.

(a) Chao1 and Shannon indices of the fecal microbiota. Each point represents an individual. Boxes indicate the median and interquartile range. (b) Spearman’s correlations (*rS* means coefficient) of the Chao1 and Shannon indices between salivary and fecal samples. (c) Principal coordinate analysis of weighted UniFrac distances. Each point represents a sample. Axes show principal coordinates, with the percentage of variance explained in parentheses. The density plots show the sample distribution along the PCo1 and PCo2 axes. The effect size (*R*^2^) was calculated by Adonis. (d) The PCo1 axis values in (c) of patients. The same participant before and after treatment was connected by a line. (e) Bar plot representing the average abundance of the top 10 genera in the fecal samples of healthy participants and patients with periodontal disease before and after treatment. The remainder were labeled ‘others’. (f) Differentially abundant genera (adjusted *P*-value <0.05) between healthy participants and pretreatment patients analyzed by ANCOMBC. (g) Changes in the featured genera in (f) before and after treatment. The same participant before and after treatment was connected by a line. (h) Spearman’s correlation of the relative abundance of salivary (*Fretibacterium* and *Cardiobacterium*) and fecal (Lachnospiraceae UCG 004 and *Intestinibacter*) genera. *p < 0.05, **p < 0.01, ***p < 0.001 with N.S. indicating no statistical significance; pairwise Adonis (C) and two-tailed paired t-test (F).

**Table 2.** Correlation between α-diversity in gut microbiota and clinical parameters.

	Chao1 index		Shannon index	
Valuables	*rho*-value	*P*-value	*rho*-value	*P*-value
PPD	0.005	0.968	0.008	0.949
CAL	0.072	0.539	0.075	0.52
BOP	0.129	0.269	0.104	0.373
PISA	0.09	0.444	0.071	0.543
PESA	− 0.005	0.968	0.019	0.874
Number of retained teeth	− 0.128	0.276	− 0.055	0.639
PCR	0.286	0.0128	0.256	0.0265

Associations between microbial alpha diversity indices and clinical parameters were assessed using Spearman’s rank correlation.

PCR = Plaque control record, PPD = Probing pocket depth, CAL = Clinical attachment level, BOP = Bleeding on probing, PESA = Periodontal epithelial surface area, PISA = Periodontal inflammatory surface area.

**Table 3.** Correlation between β-diversity in microbial composition and clinical parameters.

	Oral microbiota		Gut microbiota	
Valuables	*r*^2^-value	*P*-value	*r*^2^-value	*P*-value
PPD	0.31085665	0.001	0.0807152	0.048
CAL	0.31882944	0.001	0.08694678	0.034
BOP	0.37568063	0.001	0.02673665	0.35
PISA	0.33179242	0.001	0.05129042	0.139
PESA	0.22723145	0.001	0.08289686	0.042
Number of retained teeth	0.14292922	0.007	0.0007114	0.984
PCR	0.501264	0.001	0.02049224	0.443

Beta diversity was evaluated by principal coordinates analysis (PCoA) based on weighted and unweighted UniFrac distances. Clinical variables were fitted as vectors onto the ordination space using environmental fitting (envfit, vegan v2.6–4), and statistical significance was determined by 999 permutations.

PCR = Plaque control record, PPD = Probing pocket depth, CAL = Clinical attachment level, BOP = Bleeding on probing, PESA = Periodontal epithelial surface area, PISA = Periodontal inflammatory surface area.

Comprehensive correlation analysis of the abundance of oral and gut bacteria revealed two pairs, *Fretibacterium*, a well-known periodontal pathogen, and *Cardiobacterium*, which exhibited significant positive correlations (Figure 2h). However, no positive correlation was detected between the same genera in the oral and gut microbiota. To investigate the details of the ectopic colonization of the gut microbiota by oral bacteria, we visualized the genera and ASVs detected both in the saliva and feces. Although 18% of genera were commonly detected in the saliva and feces, this percentage decreased to 1% at the ASV level (Figure S3A). The number and cumulative abundance of the commonly detected ASVs were comparable between healthy participants and patients (Figure S3B-C). Additionally, periodontitis-associated bacteria that were increased in the saliva of the patients were rarely detected in the feces (Figure S3D). Overall, factors other than the ectopic colonization of the gut by oral bacteria may contribute to the dysbiosis of the gut microbiota in the patients, and the periodontal treatment resulted in limited improvement of dysbiosis during the follow-up period in this study.

### Characterization of the gut microbiota by co-abundance analysis

**Figure 3.**
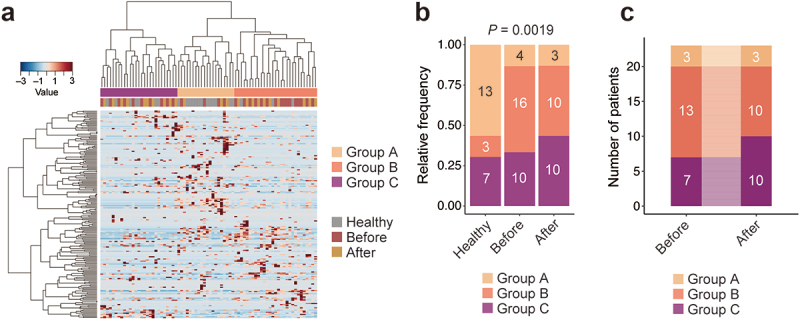
Participant clustering based on gut microbiota structures.

Although a few bacteria with different abundances between healthy participants and patients were detected (Figure 2f), it seemed difficult to characterize the gut microbiota of the patients with periodontal disease, probably due to the considerable variation in the gut microbiota between individuals (Figure S3E). Therefore, we performed hierarchical clustering to classify the participants based on their gut microbiota structure, according to a previously described co-abundance group (CAG) analysis framework [30]. For this analysis, the participants were classified into three groups (groups A to C, Figure 3a). Approximately 60% of the healthy participants were assigned to group A (Figure 3b). On the other hand, group A participants were a minor population of patients with periodontitis, both before and after treatment (Figure 3b) with the most abundant being group B before treatment. The proportions in group C were comparable between healthy participants and patients before treatment and slightly increased after treatment (Figure 3b). Periodontal treatment had only a slight impact on the gut microbiota in this analysis, with only a few patients (*N* = 3) moving from group B to group C with the treatment (Figure 3c), thus supporting the data described above (Figure 2).

(a) Hierarchical clustering of participants based on the fecal microbiota at the genus level. The participants were clustered into three groups (groups A to C) by hierarchical Ward-linkage clustering (PERMANOVA, *p* < 0.001). (b) Bar plot showing the proportion of participants clustered into each group. The number in the bar represents the number of participants in each group. The *P*-value for differences in group proportions was calculated using Fisher’s exact test. (c) Alluvial plot illustrating the intergroup transitions of patients (those with paired samples) after treatment. Numbers indicate the number of patients assigned to each group before and after treatment.

**Figure 4.**
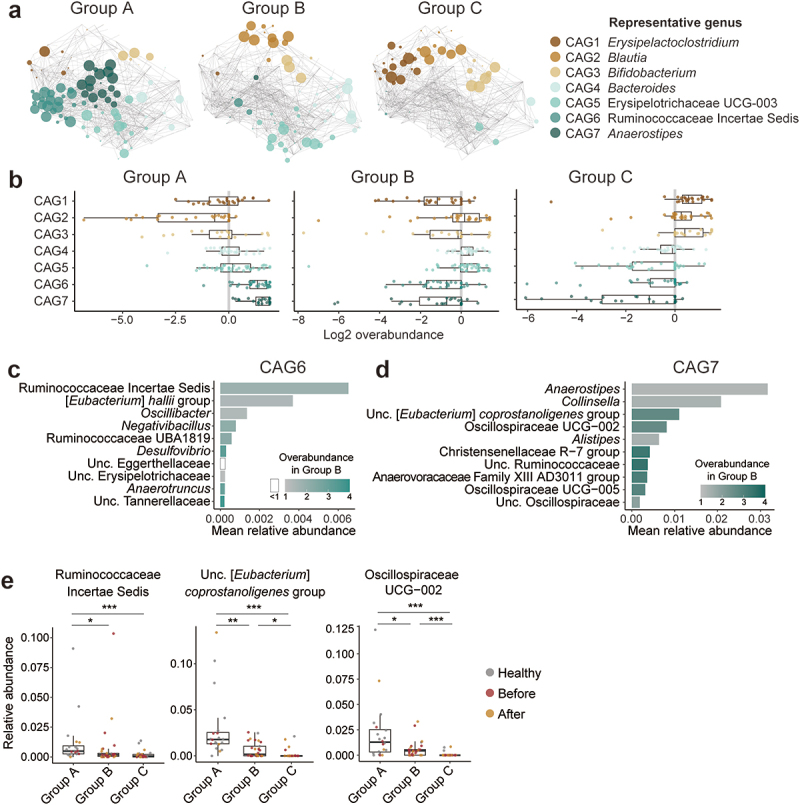
Co-abundance group analysis of the gut microbiota.

We next conducted a CAG analysis based on the previously reported method [30] to determine the characteristics of the gut microbiota in each group. Based on their co-abundance correlations, the bacterial genera were clustered into seven groups (CAG1 to 7) (Figure S4A, B). Group A, which was dominant in healthy participants (Figure 3b), exhibited increased abundance of genera belonging to CAG6 and CAG7 (Figure 4a,b). These CAGs consist of producers of short-chain fatty acids (SCFAs) such as Ruminococcaceae, *Eubacterium*, and Oscillospiraceae (Figure 4c–e). Functional prediction with Phylogenetic Investigation of Communities by Reconstruction of Unobserved States 2 (PICRUSt2) also demonstrated that pathways involved in SCFA production, such as pyruvate fermentation and L-lysine fermentation, were more enriched in group A than in group B participants (Figure S5A, B). In contrast, group B participants had a high abundance of the other genera belonging to CAG2, CAG4, and CAG5 (Figure 4a,b). Unlike those in group A, genes associated with lipid metabolism-related pathways such as palmitoleate biosynthesis and fatty acid elongation were enriched in group B.

Fecal genera were clustered into co-abundance groups (CAGs). (a) Network plots illustrating the over-abundant genera in each CAG. The color and size of each circle correspond to the CAG and over-abundance of a genus, respectively. The most abundant genus in each CAG is shown as the representative genus. (b) Each point represents a genus. Boxes indicate the median and interquartile range of log₂ over-abundance values for each participant group (groups A to C). (c, d) Mean relative abundance and the over-abundance of the top 10 genera within CAG6 (C) and CAG7 (D) in group B participants. (e) Box plots showing the relative abundances of over-abundant genera in CAG6 and CAG7 (over-abundance >1.5 and mean relative abundance > 0.5%). Each dot represents a sample and is colored according to the participant type. *p < 0.05, **p < 0.01, ***p < 0.001; Kruskal – Wallis test with Dunn’s test.

### Serum metabolome profile and its relation to the gut microbiota

**Figure 5.**
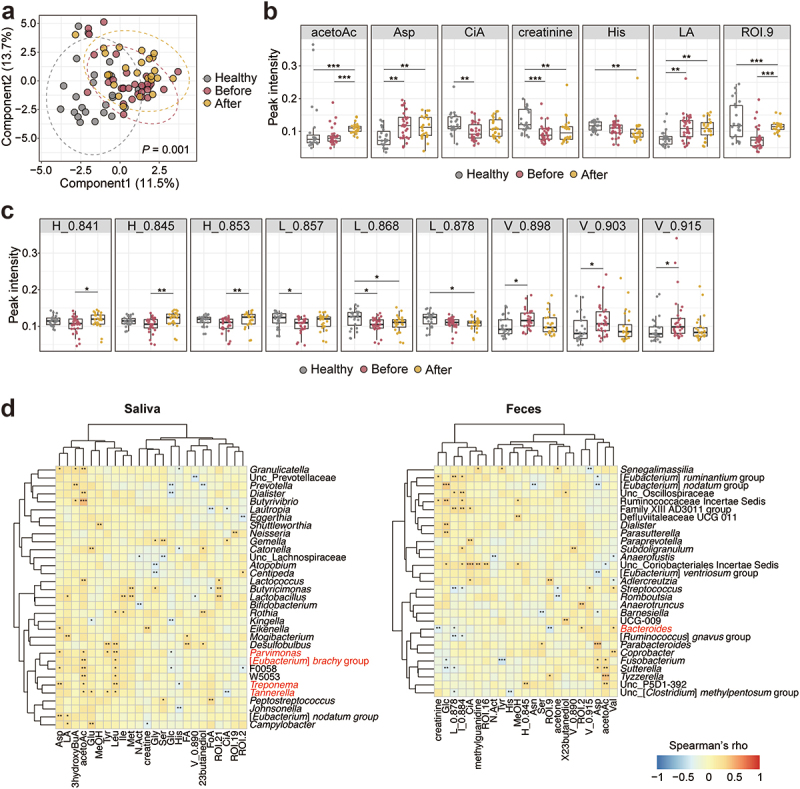
Serum metabolite profiles of healthy participants and patients with periodontal diseases.

We next quantified serum metabolites to examine the systemic effects of the altered microbiota in the patients. Partial least squares-discriminant analysis (PLS-DA) revealed that the profile of water-soluble metabolites in both pre-and posttreatment patients was distinct from that in healthy participants (Figure 5a). Individual water-soluble metabolites were differentially abundant among the groups. Interestingly, some of these metabolites were related to lipid metabolism (acetoacetate and citrate) (Figure 5b). Similarly, a differential abundance of lipoproteins was noted among the groups characterized by an increase in HDL-cholesterol and a decrease in VLDL-cholesterol after treatment in the patients (Figure 5c). We further examined the associations between serum metabolites and salivary or fecal bacteria. Among several correlated bacteria and metabolites, several periodontitis-associated bacteria (shown in red) had significant correlations with serum metabolites. In addition, the abundance of *Bacteroides*, which was increased in the patients’ feces (Figure 2e), was also positively correlated with the serum metabolites (Figure 5d).

(a) Partial least squares-discriminant analysis (PLS-DA) of serum water-soluble metabolites. Each point represents a sample. Axes show components explaining the variance between participant groups. Ellipses indicate the 95% confidence intervals for each group. The P-value was calculated by a permutation test (1,000 permutations). (b, c) Differentially abundant water-soluble metabolites (b) and lipoproteins (c) among the groups (adjusted P-value <0.05 according to the Kruskal – Wallis test). (d) Heatmaps showing Spearman’s correlation coefficients between serum metabolites and salivary (left) and fecal (right) microbiota at the genus level. The genera whose abundance significantly increased in patients (Figures 1f and 2e) are highlighted in red. *p < 0.05, **p < 0.01, ***p < 0.001; Dunn’s test (b, c) and Spearman’s correlation coefficient (d).

## Discussion

In this study, we analyzed the microbial composition of paired samples of unstimulated saliva and feces and the serum metabolite profiles of systemically healthy participants with or without generalized chronic periodontitis. We also assessed the effect of periodontal treatment on the microbiota and metabolome of the patients. We report that patients with periodontitis exhibit dysbiosis of the gut microbiota, characterized by increased alpha diversity and potential alterations in bacterial function. These changes coincided with the serum metabolite profiles.

The population we analyzed was typical of patients with chronic periodontitis, as evidenced by clinical indicators and CRP levels [43]. In addition, serum GDF15 levels, which were high before treatment, significantly decreased with treatment. GDF15 is expressed by various cell types, and its expression is induced upon cell/tissue/metabolic stress. Consequently, plasma concentrations of GDF15 are elevated in various conditions such as aging, obesity, cancer, cardiovascular disease, and autoimmune diseases [44]. Besides such systemic diseases, a causal association of immunosenescence and inflammaging with periodontitis and vice versa has only recently been acknowledged [45]. Periodontitis is not only associated with such pathology but is also known to accelerate biological aging. Persistent periodontopathic bacterial infection induces the accumulation of senescent cells in both periodontal tissues [46] and intestinal epithelial cells [47]. Although the relationship between dysbiotic gut microbiota and serum GDF15 has yet to be determined, these data suggest that elevated GDF15 could be a consequence of both oral and gut dysbiosis.

The oral cavity and lower gastrointestinal tract are connected, and sustained swallowing of saliva containing large amounts of bacteria with an unhealthy compositions, such as those observed in periodontitis, may affect the gut microbiota, which has a significant impact on systemic health. The oral microbiota is the second most dense and has comparable diversity to the gut microbiota, with distinct compositions depending on the anatomical location of the oral cavity [48]. Although dysbiosis of subgingival bacteria, where there is an increased abundance of periodontopathic bacteria which are usually very few or absent in healthy periodontium [49,50], is a characteristic feature of periodontitis, the salivary microbiota reflects the overall periodontal condition of the patients [51] and is easy to collect.

Consistent with other reports [21,49,52–54], the Chao1 index and the number of ASVs in the salivary microbiota were greater in patients with untreated periodontitis than in healthy participants and decreased after successful periodontal treatment. In addition, we demonstrated that the Shannon index increased in patients before treatment but decreased after treatment, reflecting an increase in periodontitis-associated bacteria and a reduction in the abundance of the commensal bacterium *Streptococcus*. These changes were indeed associated with periodontal inflammation. The overall salivary microbiota composition was also significantly different between healthy participants and patients and between before and after treatment. The majority of characteristic bacteria in patients’ saliva were periodontopathic bacteria, demonstrating that the study participants were appropriate. Decreased alpha diversity in the gut microbiota has been linked to inflammatory diseases [55]. However, recent studies have demonstrated that patients with age-related diseases such as Parkinson’s disease and Alzheimer’s disease show an increased Chao1 index of the gut microbiota [56,57]. Therefore, the elevated indices of richness observed in our study in patients may impact age-related diseases, although further research is needed to clarify the relationship between gut microbiota diversity and age-related diseases.

The PCoA clearly depicted the alteration of the gut microbiota in patients, although only two genera, *Bacteroides* and *Odoribacter*, were detected as significantly increased bacteria in the patients. Recently, *Odoribacter* was reported to be associated with Alzheimer’s disease [58], atherosclerosis [59], and various cancers [60,61], all of which are also known to be associated with periodontitis. CAG analysis also demonstrated that patients with periodontitis have distinct gut microbiota compared to healthy participants. Hierarchical clustering of the participants based on the gut microbiota revealed that the proportions of the group A and B microbiota could discriminate between healthy participants and patients with periodontitis. CAG analysis allowed the classification of the gut microbiota of the study participants into seven CAGs with characteristic combinations in each group. Periodontal health-associated group A was characterized by an enrichment of bacteria known to be SCFA producers, which is also supported by the functional prediction using PICRUSt2.

We also confirmed alterations in serum metabolite profiles in patients, which serve as supporting evidence of potential functional changes in the gut microbiota, as the serum metabolome profile partly reflects the gut microbiome [62]. Consistent with those in the gut microbiota, the levels of water-soluble metabolites and lipid metabolites in the serum of patients with periodontitis differed from those in the sera of healthy participants. After periodontal treatment, the number of patients belonging to group C was increased. Group C was characterized by a high abundance of CAG1, CAG2, and CAG3, in which *Erysipelactoclostridium*, *Blautia*, and *Bifidobacterium* are the representative genus, respectively. These genera are known to produce SCFA, especially acetate [63]. Acetate is involved in the regulation of glucose metabolism by inhibiting insulin signaling in adipocytes and promoting secretion of gastric inhibitory peptide in K cells via G protein-coupled receptor (GPR) 43 signaling [64].

In the metabolome analysis, histidine and acetoacetate were of particular interest. Histidine, which was decreased after periodontal treatment, is metabolized by gut microbiota to acetate, butylate, and histamine [63]. This is consistent with the increased relative abundance of SCFA-producing bacteria after periodontal treatment. On the other hand, acetoacetate was increased after periodontal treatment. Acetoacetate, a ketone body, is produced from acetyl-CoA in the liver under ketogenic conditions. Recently, it has been demonstrated that acetoacetate promoted lipid utilization in the plasma via lipolysis by plasma lipoprotein lipase activation [65]. Although the participants maintained a regular diet, making the cause of increased acetoacetate unclear, it is possible that periodontal intervention affected glucose metabolism.

Consistent with the previous studies [66], periodontitis was associated with lower HDL-cholesterol and higher LDL-cholesterol, which is associated with increased risk of cardiovascular diseases. These lipid profiles improved after periodontal treatment. While the precise mechanisms remain unknown, alteration of the structure of the gut microbial community, reduction of systemic inflammation, and the change of the metabolic profiles discussed above may be involved.

The oral-gut axis represents a potential mechanism linking periodontal disease with systemic diseases [67]. Alpha diversity indices, such as the Chao1 and Shannon indices, were positively correlated between the oral and gut microbiota. In addition, these indices were positively correlated with PCR values, which represent the bacterial load in the oral cavity. The increase in alpha diversity in patients’ saliva was attributed to the increase in periodontitis-associated bacteria; however, these bacteria were not detected in the feces of the patients. Furthermore, unlike in previous studies [68,69], the number of genera and ASVs commonly found between saliva and feces was very small in our study. Also, beta diversity in patients’ feces was correlated with PPD, CAL, and PESA, the severity indexes of periodontitis but not with PCR. These findings suggest that gut dysbiosis in patients might have been induced irrespective of ectopic colonization by oral bacteria. In this regard, we have demonstrated that oral gavage of *P. gingivalis* alters the gut microbiota without causing colonization in mice [10], and this finding has recently been confirmed [70]. Given the distinct composition between the luminal microbiota represented by feces and mucosa-associated microbiota [71,72], we cannot rule out the possibility that the oral microbiota colonized the mucosa-associated area and was, therefore, barely detected in the feces. However, further experiments are needed to elucidate the mechanism by which periodontitis induces gut dysbiosis.

Unlike that of the oral microbiota, the increase in alpha diversity of the gut microbiota in patients was not reversed after treatment. PCoA and CAG analysis also demonstrated that treatment has limited effects on the structure of the gut microbiota of patients. In contrast to our findings, a recent study by Baima et al. revealed that periodontal treatment partially restored alterations in the gut microbiota of patients [68]. In our study, we sampled samples at 1–3 months and Baima et al. sampled samples at 3 months after treatment, which is a common timeframe for evaluating the effects of periodontal treatment [73]. These data imply that there may be a time lag of several months for periodontal treatment to contribute to the improvement of the gut microbiota, and a time course analysis of the gut microbiota after treatment could address this possibility. Additionally, our data suggest that interventions targeting the gut microbiota, particularly the administration of SCFA-producing bacteria, along with periodontal treatment may assist in recovery from gut dysbiosis.

Given the compelling evidence that gut dysbiosis induced by experimental periodontitis in animals induces deterioration of periodontitis-related pathologies, this area of research has drawn great attention. However, human studies have just started and there are no consistent findings [21]. We have, for the first time, elucidated the details of the oral-gut axis in Asian patients with periodontal disease. As the structure of the gut microbiota varies among different ethnicities, the frequency of oral bacteria detected in the fecal samples differed between our study and others [21], although gut dysbiosis in patients was similarly observed. In addition, we have shown not only taxonomical differences but also potential changes in the function of the gut microbiota and their effects on systemic metabolite profiles in patients. These findings underscore the importance of functional analyses of the gut microbiota in patients to clarify connections between periodontitis and other intestinal, systemic, and age-related diseases.

**Figure 6.**
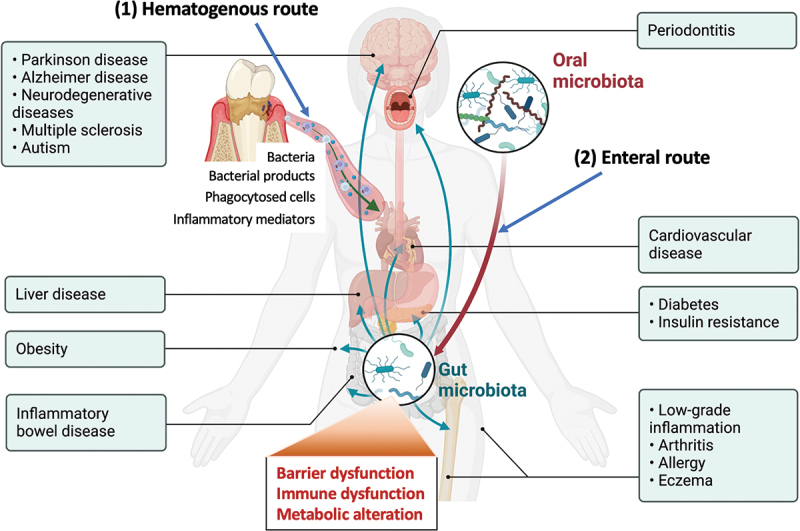
The possible two pathways connecting periodontitis and systemic diseases.

In this study, we highlighted the potential impact of swallowed oral bacteria on the dysbiosis of gut microbiota, which may in turn affect host physiology. However, several limitations should be acknowledged. In addition to the enteral route, the hematogenous route, through which bacteria and/or bacterial products may disseminate via the bloodstream is also an important pathway that can modulate systemic inflammation and immune function [74] (Figure 6). Therefore, further research is needed to relative contribution of the enteral route to systemic health outcomes. Also, it remains to be elucidated whether periodontitis-associated alterations in the gut microbiota can indeed induce or deteriorate systemic diseases. Thus, it seems necessary to conduct experiments where fecal samples from patients are transplanted into disease model mice, in addition to the conventional approach of administering periodontal bacteria or patients’ saliva to mice (10, 26).

(1) Heamtogenous route: Dysbiotic oral bacteria, bacterial products, phagocytosed cells, and/or inflammatory mediators get into the systemic circulation and reach various organs and tissues via systemic circulation and induce inflammatory changes.

(2) Enteral route: Dysbiotic bacteria reach the lower intestinal tract and mediate barrier dysfunction, immune dysfunction, and metallic alteration resulting in systemic tissue damage.

The figure is created with Biorender.com.

In conclusion, we provide evidence that patients with periodontitis exhibit gut dysbiosis and alterations in serum metabolite profiles regardless of ectopic colonization by pathogenic oral bacteria. Our results suggest that periodontal treatment may not be highly effective at improving the gut microbiota, highlighting the importance of interventions targeting the gut microbiota in addition to oral care.

## Supplementary Material

Supplementary Figures.docx

Table_S1_for_submission_R1.xlsx

## Data sharing

The raw sequencing data of the 16S rRNA gene are available in the DDBJ Sequence Read Archive under the accession number DRA016597 [https://ddbj.nig.ac.jp/resource/bioproject/PRJDB16140]. Subject data collected in this study, such as oral examination and metabolome data sets generated during and/or analyzed during the current study are available from the corresponding author on reasonable request.
