# Supplementary material for: Patients with periodontitis exhibit persistent dysbiosis of the gut microbiota and distinct serum metabolome
Source: J Oral Microbiol. 2025 May 8;17(1):2499284. doi: 10.1080/20002297.2025.2499284 (PMC12064113; doi:10.1080/20002297.2025.2499284)
Supplement: Supplementary Figures.docx [file ZJOM_A_2499284_SM7381.docx]

**Supplementary Information**

**Patients with periodontitis exhibit persistent dysbiosis of the gut microbiota and distinct serum metabolome**

Eiji Miyauchi, Kyoko Yamazaki, Yuuri Tsuboi, Takako Nakajima, Shigeru Ono, Kentaro Mizuno, Naoki Takahashi, Kentaro Imamura, Hidetoshi Morita, Nobuaki Miura, Shujiro Okuda, Jun Kikuchi, Nobuo Sasaki, Hiroshi Ohno, and Kazuhisa Yamazaki


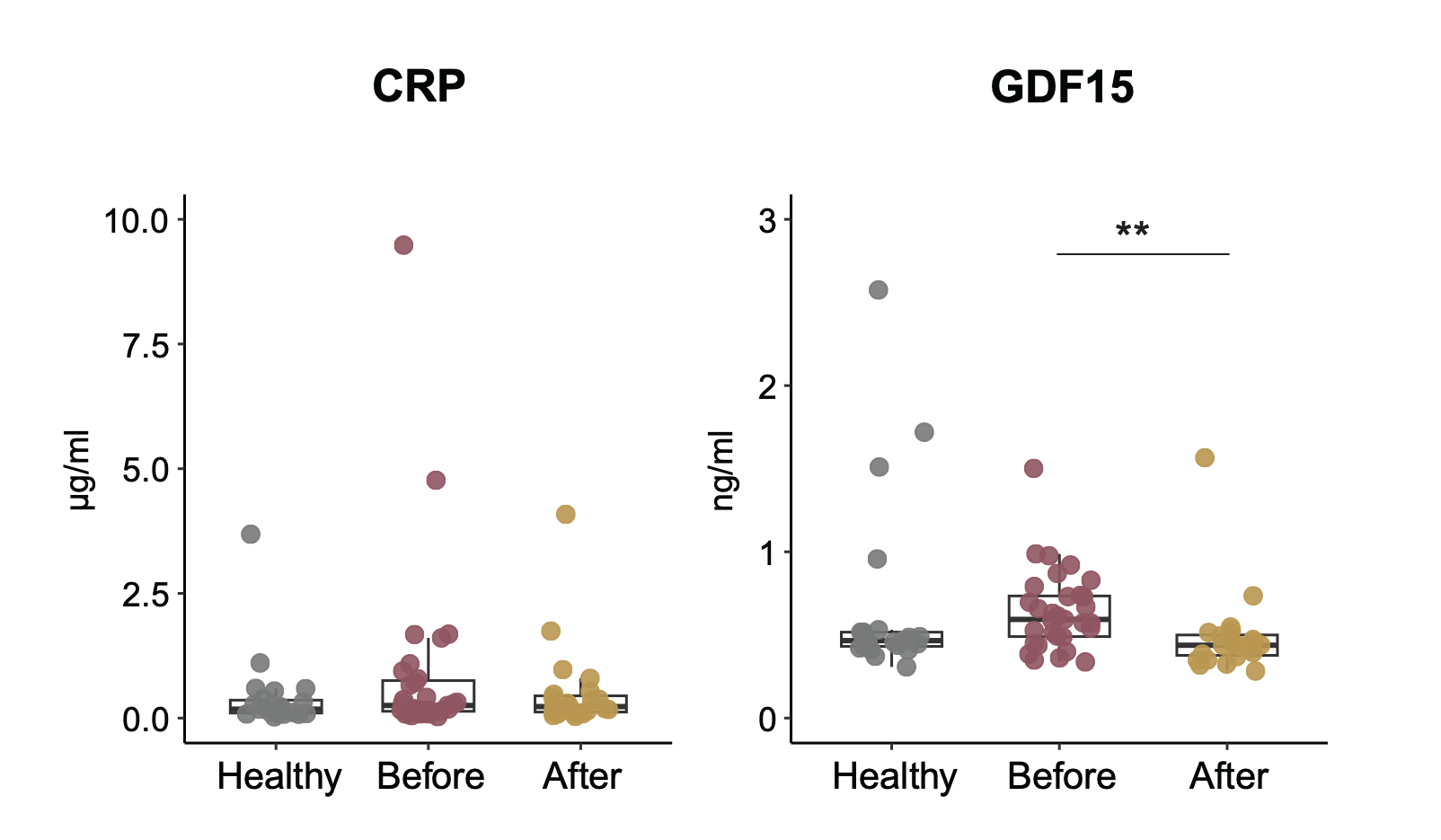


Figure S1. Serum markers of the participants

Serum CRP and GDF levels were analyzed with Human Magnetic Luminex®. **P < 0.01, Kruskal–Wallis test with Dunn’s multiple comparisons.


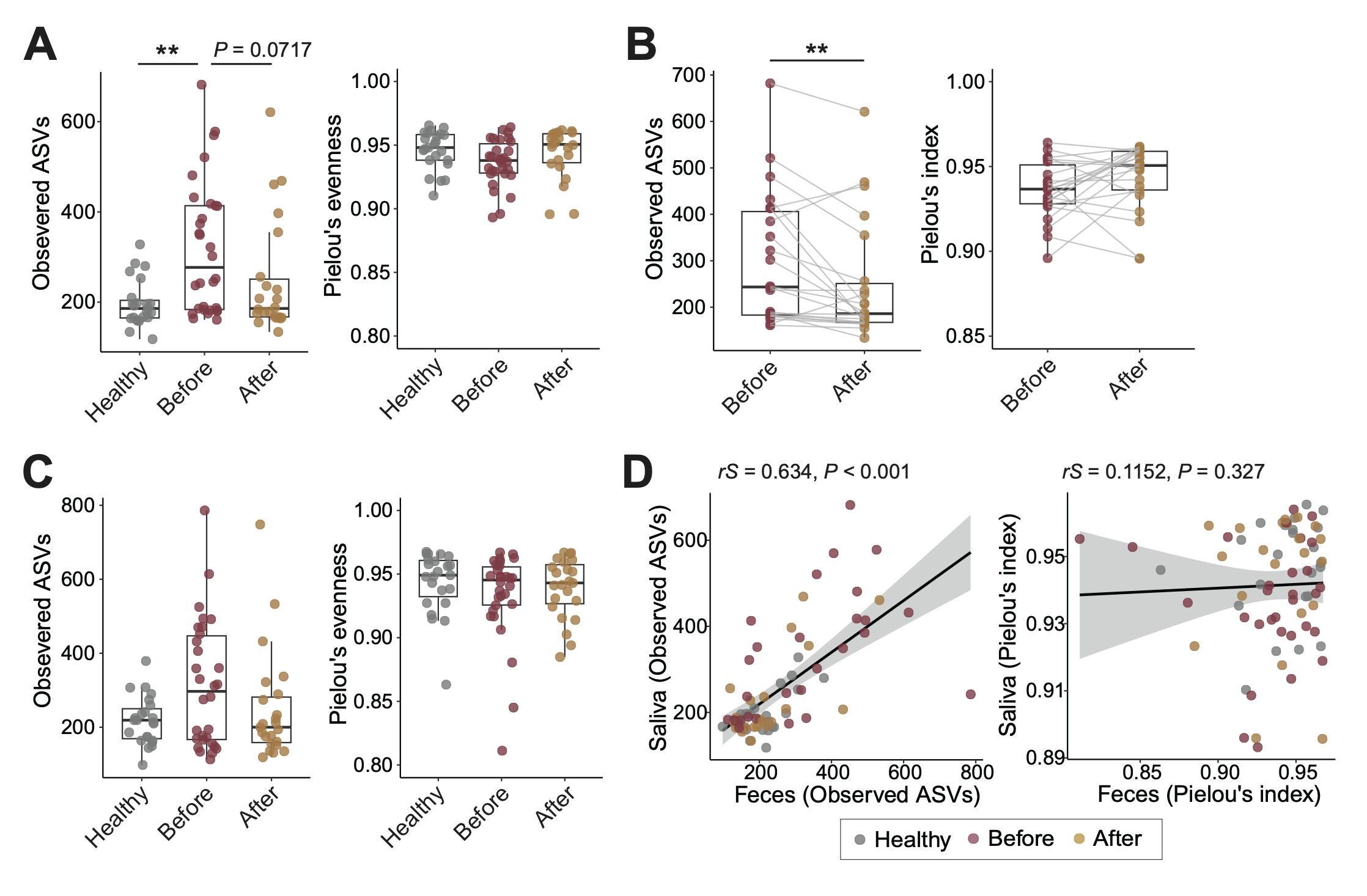


Fig. S2. Alpha diversity of oral and gut microbiota.

(A, B) The number of observed ASVs and Pielou's index of the salivary microbiota (A). The same participant before and after treatment was connected by a line (B). (C) The number of observed ASVs and Pielou's index of the fecal microbiota. (D) Spearman's correlations of the number of observed ASVs and Pielou's index between salivary and fecal samples. **P < 0.01; Kruskal–Wallis test with Dunn’s multiple comparisons (A, C) and two-tailed paired t-test (B).


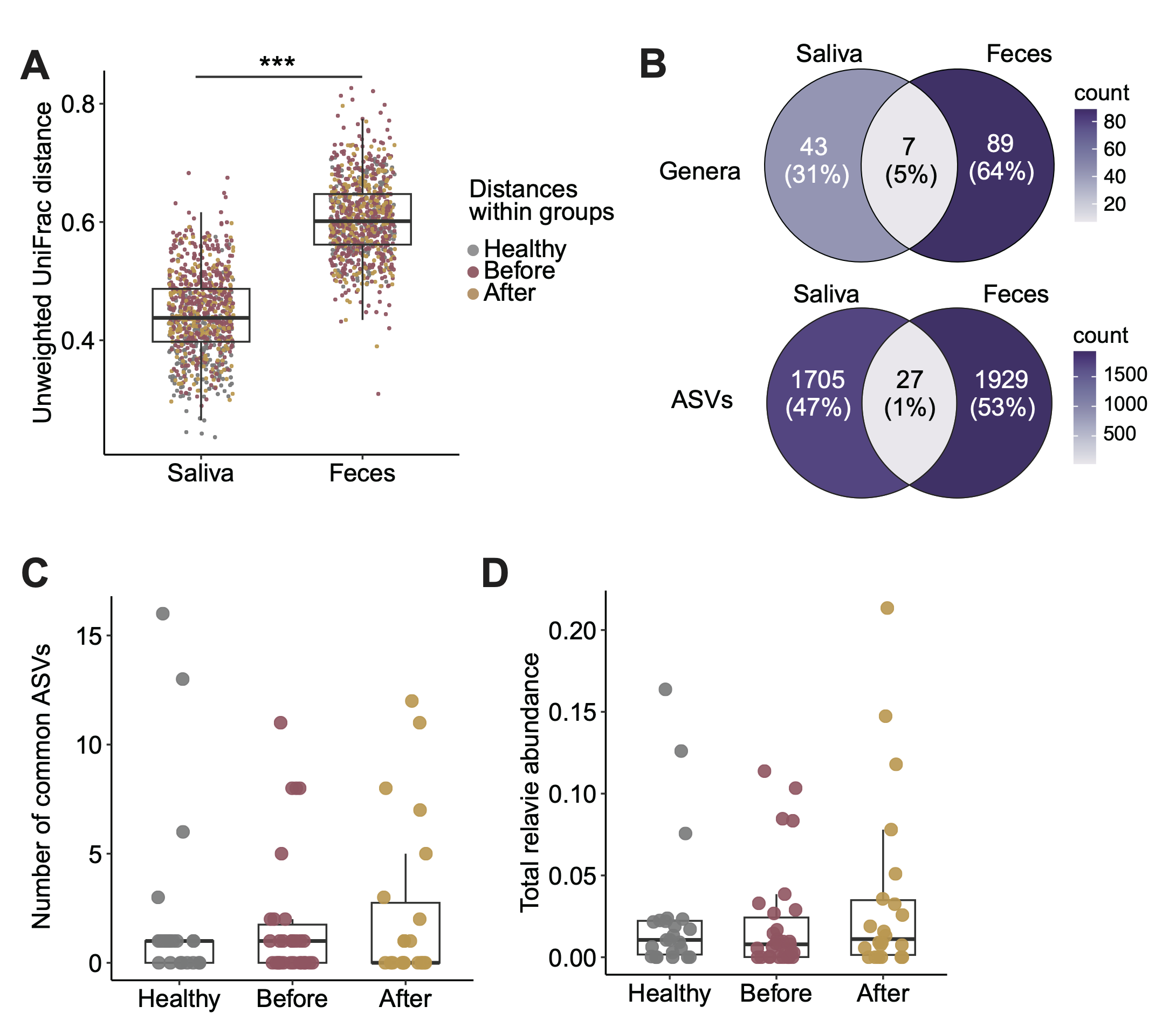


Figure S3. Variance of oral and gut microbiota

(A) Unweighted UniFrac distances within each group. ***P < 0.001, Mann–Whitney test. (B-D) Commonly detected ASVs in the saliva and fecal samples (mean abundance > 0.01%) are shown in the Venn diagram (B). Number (C) and total relative abundance (D) of these ASVs within participants.


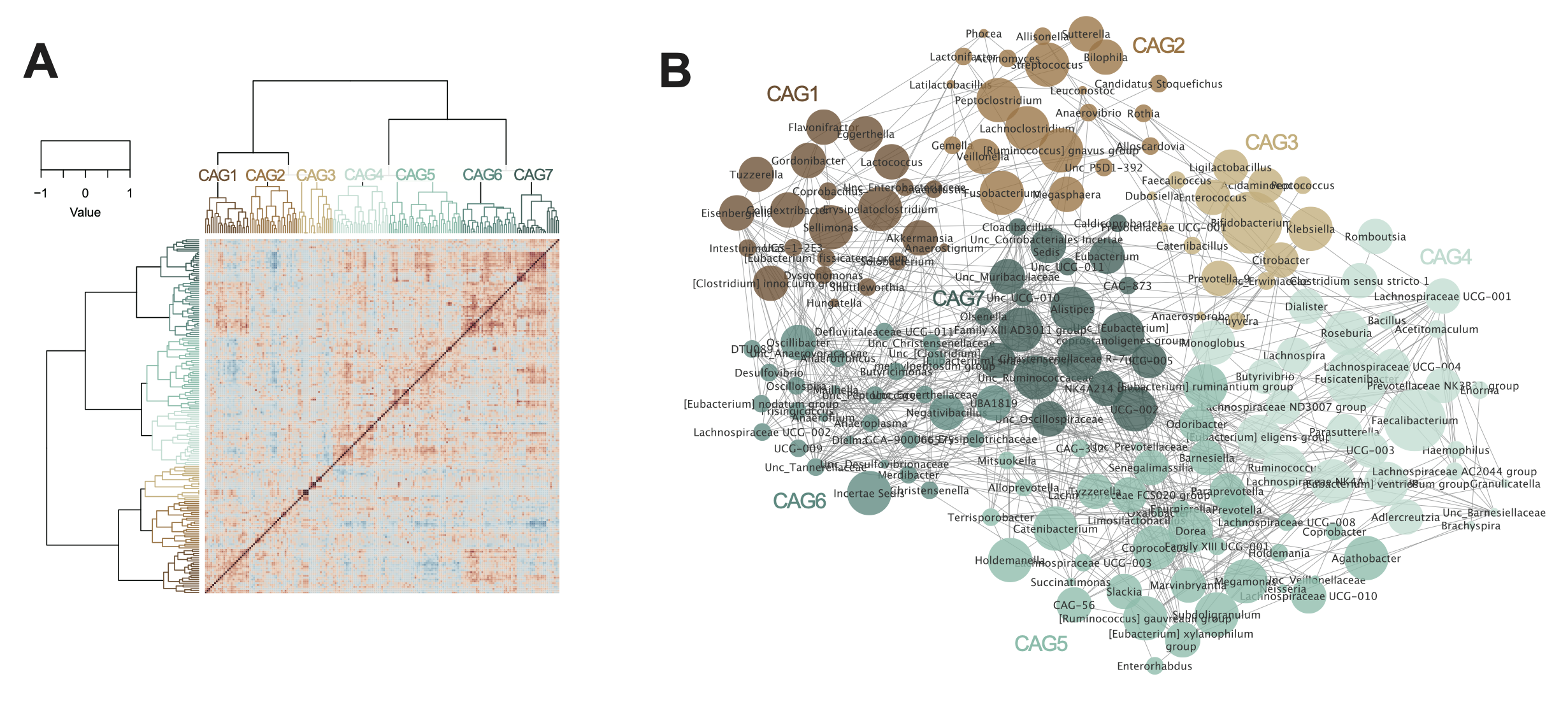


Figure S4. Clustering of genera for co-abundance group analysis

(A) Hierarchical clustering of genera based on Kendall’s tau coefficients. The genera were clustered into seven groups (CAG1 to 7) by Ward-linkage clustering (PERMANOVA, P < 0.001). (B) Network plots illustrating genera belonging to each CAG. The color and size of each circle correspond to the CAG and mean abundance of a genus, respectively.


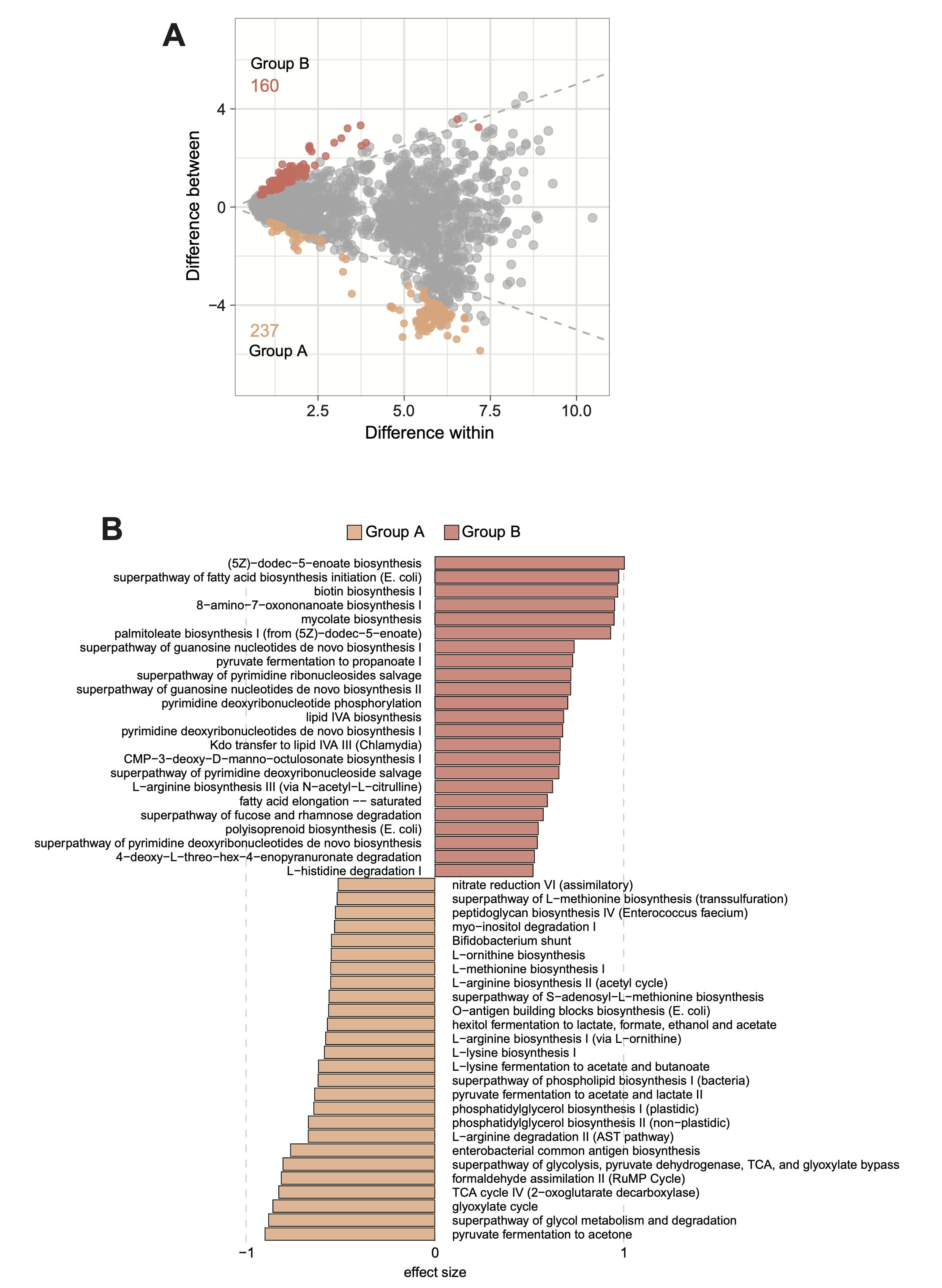


Figure S5. Functional prediction of gut microbiota

Functional information of gut microbiota was inferred with 16S rRNA gene data using PICRUSt2. (A) MA plot showing the differentially abundant KEGG orthologs between group A and B participants. (B) Differentially abundant pathways (FDR < 0.05, absolute effect size > 0.5) between group A and B participants.
